# Motifs in Brain Networks

**DOI:** 10.1371/journal.pbio.0020369

**Published:** 2004-10-26

**Authors:** Olaf Sporns, Rolf Kötter

**Affiliations:** **1**Department of Psychology and Programs in Cognitive and Neural Science, Indiana UniversityBloomington, IndianaUnited States of America; **2**C. and O. Vogt Brain Research Institute and Institute of Anatomy II, Heinrich Heine UniversityDüsseldorfGermany

## Abstract

Complex brains have evolved a highly efficient network architecture whose structural connectivity is capable of generating a large repertoire of functional states. We detect characteristic network building blocks (structural and functional motifs) in neuroanatomical data sets and identify a small set of structural motifs that occur in significantly increased numbers. Our analysis suggests the hypothesis that brain networks maximize both the number and the diversity of functional motifs, while the repertoire of structural motifs remains small. Using functional motif number as a cost function in an optimization algorithm, we obtain network topologies that resemble real brain networks across a broad spectrum of structural measures, including small-world attributes. These results are consistent with the hypothesis that highly evolved neural architectures are organized to maximize functional repertoires and to support highly efficient integration of information.

## Abstract 2

Analysis of characteristic patterns of connectivity in neuroanatomical datasets suggests that nervous systems evolved to maximize functional repertoires and support highly efficient integration of information

## Introduction

The complex vertebrate brain has evolved from simpler networks of neurons over a time span of many millions of years. Brain networks have increased in size and complexity (Jerison 1973; Butler and Hodos 1996; Kaas 2000; Krubitzer 2000), as have the flexibility of interactions with the environment and the range of potential behaviors that can be generated (Changizi 2003). Most of the rules governing the evolutionary process toward more complex brains are still unknown, although the central roles of modularization (Kaas 2000), conservation of wiring length (Cherniak 1994; Chklovskii et al. 2002), and of the elaboration of network connectivity (Laughlin and Sejnowski 2003) are becoming increasingly evident.

Systematic investigations of neuronal connectivity in the nematode Caenorhabditis elegans (White et al. 1986) and of large-scale interregional pathways in the mammalian cerebral cortex of rat (Burns et al. 2000), cat (Scannell et al. 1995; Scannell et al. 1999; Hilgetag et al. 2000; Kötter and Sommer 2000), and macaque monkey (Felleman et al. 1991; Young 1993; Hilgetag et al. 2000; Stephan et al. 2000) have demonstrated that the topology of these networks is neither entirely random nor entirely regular. Instead, analysis of structural and functional data has shown (Hilgetag et al. 2000; Sporns et al. 2000; Stephan et al. 2000; Sporns and Zwi 2004) that these networks can be characterized by a high degree of clustering, with short path lengths linking individual components, thus exhibiting small-world properties (Watts and Strogatz 1998; Watts 1999) as do many other complex networks (Strogatz 2001; Albert and Barabasi 2002). These structural attributes are instrumental in generating functional specialization (Zeki 1978; Passingham et al. 2002) and functional integration (Bressler 1995; Tononi et al. 1998; McIntosh 2000; Varela et al., 2001; Friston 2002), and they support a large repertoire of complex and metastable dynamical states (Bressler and Kelso 2001; Sporns and Tononi 2002; Sporns 2004). Fluctuating and distributed patterns of dynamical interactions among functionally specialized areas result in rapid switches in functional and effective connectivity (McIntosh et al. 1999; Büchel and Friston 2000; McIntosh et al., 2003; Brovelli et al. 2004). The structural and functional anatomy of brain networks reflects the dual challenges of extracting specialized information and integrating the information in real time (Tononi and Sporns 2003).

What rules underlie the organization of the particular types of networks that we see in complex brains? It is likely that, as networks become more complex, already existing simpler networks are largely preserved, extended, and combined, while it is less likely that complex structures are generated entirely de novo. One hypothesis states that complex and highly evolved networks arise from the addition of network elements in positions where they maximize the overall processing power of the neural architecture. This could be achieved by increasing the number of existing processing configurations or by introducing new processing configurations that add to the robustness or range of cognitive and behavioral repertoires. We may gain insight into the rules governing the structure of complex networks by investigating their composition from smaller network building blocks. Those building blocks are called “motifs” (in analogy to driving elements that are elaborated in a musical theme or composition), and they have been examined in the context of gene regulatory, metabolic, and other biological and artificial networks (Milo et al. 2002; Milo et al. 2004). Motifs occur in distinct motif classes that can be distinguished according to the size *(M)* of the motif, equal to the number of nodes (vertices), and the number and pattern of interconnections. For a more formal definition of motifs and related concepts, see Materials and Methods.

**Figure 1:**
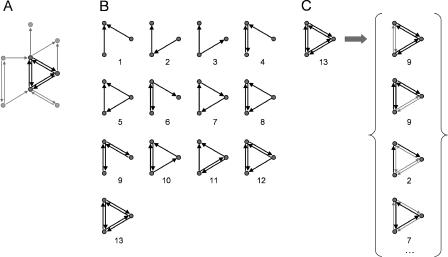
Definition of Structural and Functional Motifs, and Motif Detection (A) From a network, we select a subset of three vertices and their interconnections, representing a candidate structural motif. (B) The candidate motif is matched to the 13 motif classes for motif size *M* = 3. Numbers refer to the ID. The candidate motif is detected as a motif with ID = 13. In detecting structural motifs, only exact matches of candidate motif and motif class are counted. (C) A single instance of a structural motif contains many instances of functional motifs. Here, a structural motif (*M* = 3, ID = 13) is shown to contain, for example, two distinct instances of the functional motif ID = 9, one motif ID = 2, and one motif ID = 7. Many other distinct instances of functional motifs are present that are not shown in the figure. Note that, in order to be counted as a functional motif of size *M* = 3, all three vertices of the original structural motif must participate. For a very similar distinction between structural and functional motifs (“interlaced circuits”) and an illustration see Ashby (1960), p. 53.

While the most common definition of network motifs is based on their structural characteristics (Milo et al. 2002), structural motifs of neuronal networks form the physical substrate for a repertoire of distinct functional modes of information processing. In brain networks, a structural motif may consist of a set of brain areas and pathways that can potentially engage in different patterns of interactions depending on their degree of activation, the surrounding neural context or the behavioral state of the organism. Thus, we propose a distinction between structural and functional motifs. Structural motifs quantify anatomical building blocks, whereas functional motifs represent elementary processing modes of a network (Figure 1). In this paper, functional motifs refer to specific combinations of nodes and connections (contained within structural motifs) that may be selectively recruited or activated in the course of neural information processing. Sorting all possible structural motifs within a network as a function of motif class yields a motif frequency spectrum that records the number of distinct motifs in each structural motif class. Given the motif frequency spectrum, one can easily obtain the motif number, defined as the total number of distinct occurrences of any motif of size *M,* and the motif diversity, defined as the number of classes that are represented within the network by at least one example.

Clearly, the number of vertices *(N)* and edges *(K)* within a large network has a strong effect on the motif number and diversity of its constituent structural and functional motifs. But even if *N* and *K* are held constant, different connection patterns will result in different repertoires of such network motifs, expressed in terms of both number and diversity. These considerations lead us to formulate hypotheses concerning the rules for brain network organization in terms of network motifs. We hypothesize that neuronal networks have evolved such that their repertoire of potential functional interactions (functional motifs) is both large and highly diverse, while their physical architecture is constructed from structural motifs that are less numerous and less diverse. A large functional repertoire facilitates flexible and dynamic processing, while a small structural repertoire promotes efficient encoding and assembly.

We investigate this hypothesis first by performing an analysis of structural and functional motifs in various brain networks. We compare the motif properties of real brain networks with random networks and with networks that follow specific connection rules such as neighborhood connectivity (lattice networks). We identify some motif classes that occur more frequently in real brain networks, as compared to random or lattice topologies. Second, by rewiring random networks and imposing a cost function that maximizes functional motif number, network topologies are generated that resemble real brain networks across a broad spectrum of structural measures, including small-world attributes. The results of our analyses are consistent with the hypothesis that complex brain networks maximize functional motif number and diversity while maintaining relatively low structural motif number and diversity.

## Results

### Motif Frequency Analysis

We obtained complete structural motif frequency spectra for large-scale connection matrices of macaque visual cortex, macaque cortex, and cat cortex, for motifs sizes of *M* = 2, 3, 4, and 5 (estimations). In addition, we obtained motif frequency spectra for the matrix of interneuronal connections (“chemical synapses”) of C. elegans, for motif sizes *M* = 2, 3, and 4 (estimations). For each neural connectivity matrix we generated equivalent *(N, K)* random and lattice matrices, preserving degree distributions (*n* = 100; see Materials and Methods), and we obtained their structural motif frequency spectra for comparison. Thus, statistical significance of a motif can only be reached if it occurs in significantly increased proportions with respect to both random and lattice reference cases.

Table 1 summarizes the data for structural and functional motif number. Large-scale connection matrices exhibit a consistent statistical trend. Their structural motif number is relatively low, and their functional motif number is relatively high, with both measures approaching the corresponding values of lattice networks. All of these brain networks contain a very high proportion of connected motifs (e.g., 53.2% for *M* = 3 in macaque visual cortex versus 24.6% in corresponding random networks). All neuronal networks (all cortical networks and C. elegans) showed maximal functional motif diversity for all motif sizes examined (values of 2, 13, 199, and 9,364 for *M* = 2 to 5). Their structural motif diversity tended to be submaximal. For example, the structural motif diversity of macaque cortex was significantly reduced in comparison to random matrices (168, compared to 198 ± 1 for random networks at *M* = 4). This tendency was especially pronounced for higher values of *M* (e.g., 3,697 for macaque visual cortex, compared to 8,887 ± 112 for random networks at *M* = 5).

**Table 1:**
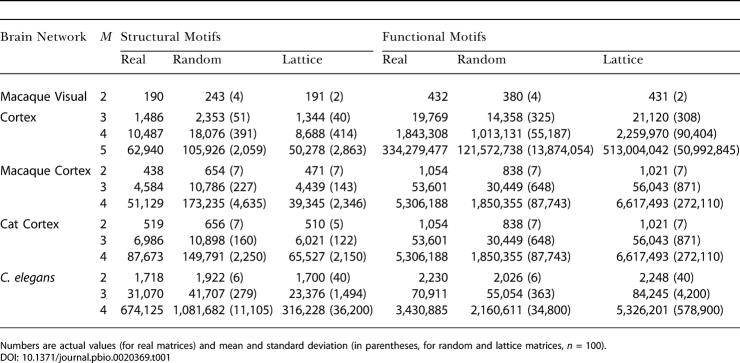
Structural and Functional Motif Number for Cortical Connection Matrices and Corresponding Random and Lattice Matrices

Numbers are actual values (for real matrices) and mean and standard deviation (in parentheses, for random and lattice matrices, *n* = 100)

**Figure 2:**
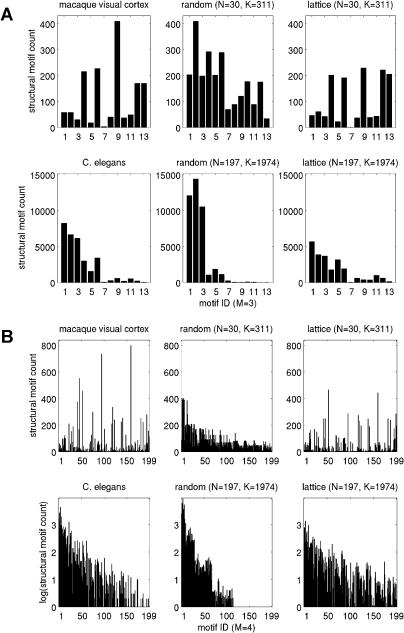
Comparison of Structural Motif Frequency Spectra for Macaque Visual Cortex and C. elegans (A) Spectra for structural motifs of size *M* = 3. (B) Spectra for structural motifs of size *M* = 4.

**Figure 3:**
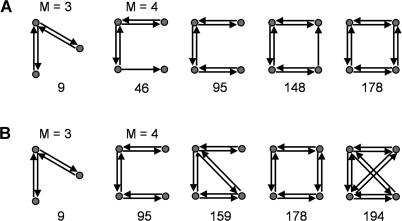
Structural Motifs that Occurred in Significantly Increased Numbers at Motif Sizes *M* = 3 and *M* = 4 (A) Structural motifs found in all three large-scale cortical networks analyzed in this study (see Table 2). (B) Structural motifs found in networks optimized for functional motif number (see Table 4). Numbers refer to the motif's ID.

Figure 2 shows motif frequency spectra for structural motifs (*M* = 3, *M* = 4) found within the network of the macaque visual cortex and C. elegans and their corresponding reference cases. Spectra of macaque and C. elegans networks are both less similar to random networks than to lattice networks. For *M* = 3, in the case of the macaque visual cortex, some motif counts appear decreased over random networks (e.g., motif identity number [ID] = 1,…,6) while other motif counts appear increased (e.g., ID = 9) over both random and lattice networks. Table 2 and Figure 3A summarize structural motifs whose motif counts were significantly increased in brain networks as compared to both random networks and lattice networks of identical degree distributions, for sizes *M* = 2, 3, and 4. Given motif frequencies from samples of *n* = 100 random or lattice networks, we calculated *z*-scores for the corresponding motifs in neuronal networks. Only structural motifs that were significantly increased (*z* > 5.0, *p* < 0.0001) in real networks as compared to both random and lattice networks are tabulated. Despite variations in size, areal composition, species, and collating authors, specific motif classes consistently emerged across several different cortical networks. Figure 3 displays those structural motifs that were consistently encountered in all three cortical connection matrices. Particularly noteworthy is the consistent appearance of motif ID = 9 (*M* = 3) in all cortical matrices examined in this study. The appearance of this motif cannot be explained by a higher proportion of reciprocal (mutual) edges (a motif of size *M* = 2): While random networks contain fewer such edges, lattice networks contain an equally high proportion of such edges (for example, macaque visual cortex has 69 single edges and 121 double edges, while a sample of 100 comparison lattice networks contains 70.6 ± 4.71 single edges and 120.2 ± 2.36 double edges). No motif of size *M* = 2 was significantly increased in frequency for any of the connection matrices in this study (Table 2). Furthermore, other motifs containing double edges (e.g., ID = 6, 12, etc.) were not increased. A different set of significantly increased structural motifs was found for C. elegans. Motif ID = 9 was not significantly increased in frequency, while two other non-connected motifs (ID = 4 and 6) occurred more frequently than expected.

**Table 2:**
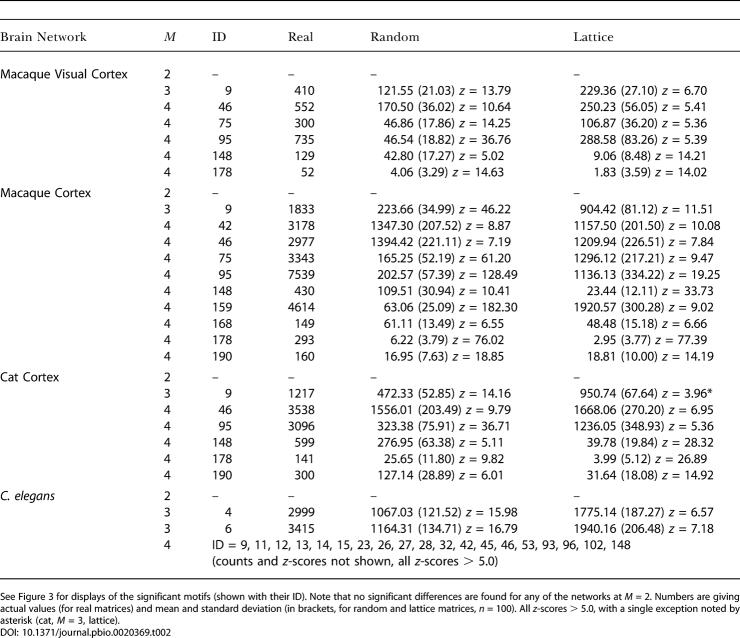
Structural Motifs That Are Significantly Increased in Brain Networks

See Figure 3 for displays of the significant motifs (shown with their ID). Note that no significant differences are found for any of the networks at *M* = 2. Numbers are giving actual values (for real matrices) and mean and standard deviation (in brackets, for random and lattice matrices, *n* = 100). All *z*-scores > 5.0, with a single exception noted by asterisk (cat, *M* = 3, lattice)

**Table 4:**
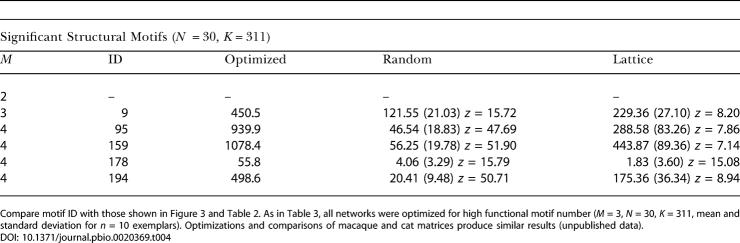
Significantly Increased Structural Motifs of Optimized Networks

Compare motif ID with those shown in Figure 3 and Table 2. As in Table 3, all networks were optimized for high functional motif number (*M* = 3, *N* = 30, *K* = 311, mean and standard deviation for *n* = 10 exemplars). Optimizations and comparisons of macaque and cat matrices produce similar results (unpublished data)

**Table 3:**
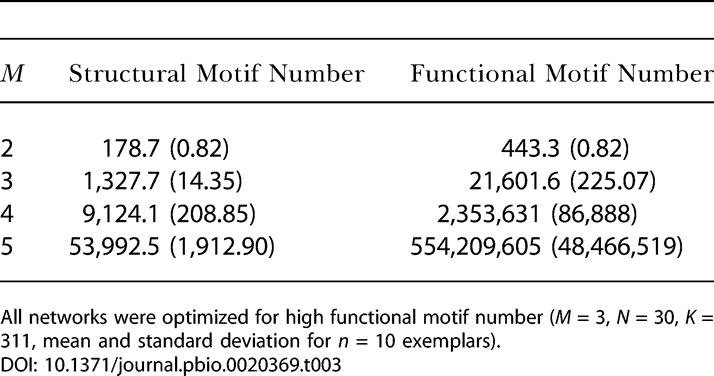
Structural Motif Number and Networks Optimized for Functional Motif Number

All networks were optimized for high functional motif number (*M* = 3, *N* = 30, *K* = 311, mean and standard deviation for *n* = 10 exemplars)

Each vertex (brain area) participates in a subset of the structural motifs that compose the entire network. We asked whether individual brain areas participate in similar or different sets of motifs and whether motif participation might reveal functional relationships. We define the motif fingerprint of a brain area as the number of distinct structural motifs of size *M* that the area participates in. Motif fingerprints characterize brain areas, as do other structural and functional features (Passingham et al. 2002), and they are directly related to other connectional metastructures forming various kinds of network participation indices (Kötter and Stephan, 2003).

**Figure 4:**
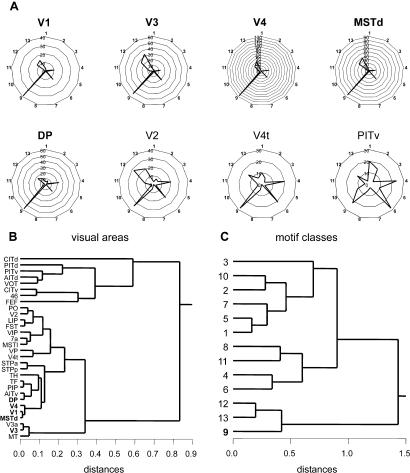
Motif Fingerprints for Motif size *M* = 3 in Macaque Visual Cortex (A) Motif fingerprints for five areas with significantly increased motif ID = 9 (V1, V3, V4, MSTd, DP, names in bold) as well as areas V2, V4t, and PITv. Polar plots display the motif participation number for 13 motif classes with *M* = 3 (see Figure 1). Note that, despite differences in the absolute motif participation numbers, areas V1, V3, V4, MSTd and DP show highly similar motif fingerprints. (B) Hierarchical cluster analysis of motif fingerprints. The Pearson correlation coefficients between all pairs of motif fingerprints were used in a consecutive linking procedure using Euclidean distances based on the farthest members of each cluster (for details see Kötter and Stephan [2003]). Areas with more similar motif fingerprints are linked at smaller distances. The five areas with significantly increased motif ID = 9 are indicated in bold typeface. (C) Hierarchical cluster analysis of single area motif frequency spectra using the same procedures on orthogonal data of (B). Motif classes 9, 12, and 13 covary across the 30 visual areas and form a distinct branch of the cluster tree.

Figure 4 shows polar plots of motif fingerprints (*M* = 3) for several visual areas of macaque visual cortex. Motif ID = 9 was the only motif found to be significantly increased over both random and lattice networks, but it was increased for only five visual areas (V1, V3, V4, MSTd, and DP). All of these areas showed highly similar motif fingerprints characterized by a specific ratio of motif classes 9, 12, and 13 (Figure 4A and 4C). Other areas, such as V2, V4t, and PITv show very different motif fingerprints (Figure 4A), and cluster analysis reveals them as members of clusters of visual areas participating in a different set of motifs. For example, most inferotemporal areas as well as visually related prefrontal areas 46 and FEF belong to a separate cluster with motif fingerprints that differ significantly from those of all other cortical areas (Figure 4B).

### Optimization of Motif Number

We hypothesized that high functional motif number and diversity represent important ingredients in the global organization of cortical networks, and that a selective advantage for these two properties might contribute to the generation of other significant structural properties. To test this hypothesis we applied an evolutionary algorithm (Sporns et al. 2000) that selects for networks with high functional motif number, while rewiring their connectivity. All simulations were carried out with networks of size *N* = 30, *K* = 311 (matching macaque visual cortex), in generations of 10 individuals, with a low rewiring rate of one connection per generation and a survivor rate of one network per generation, over 2,000 generations. Convergence was robust and consistent structural features of optimized connection matrices were observed.

**Figure 5:**
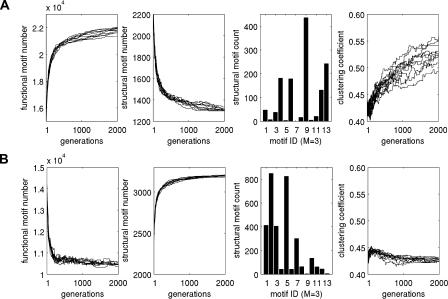
Properties of Networks (*n* = 10) Optimized for Structural and Functional Motif Number (A) Maximization of functional motif number (*N* = 30, *K* = 311). Each maximization starts from different random initial conditions, including a different set of 10 random networks. From left to right, each graph shows plots of functional motif number, structural motif number, motif frequency spectrum (*M* = 3) of optimized networks, and clustering coefficient. (B) Maximization of structural motif number (*N* = 30, *K* = 311). Graphs are as in (A). Compare the motif frequency spectrum in (A) with the corresponding plot for the macaque visual cortex in Figure 2A (first row, left bar graph). Initially, random networks in generation 1 exhibited frequency spectra identical to those for random networks in Figure 2A (first row, middle panel).

Figure 3B, Figure 5, Table 3, and Table 4 summarize results obtained from the optimizations. When maximizing functional motif number (Figure 5A), we obtained networks that closely resembled real brain networks with respect to their structural and functional motif number, motif diversity (unpublished data), structural motif frequency spectrum, and the specific structural motifs that occurred with significantly increased frequency (Tables 3 and 4). Optimizing functional motif number invariably resulted in a significant decline in the number of structural motifs. Figure 3B illustrates the set of structural motifs that appeared in significantly increased numbers after optimizing functional motif number. Note the appearance of motifs that are identical or highly similar to those obtained from an analysis of large-scale cortical matrices. These structural similarities are observed for the motif size at which the networks were optimized (*M* = 3) as well as at lower and higher motif sizes (Table 4). In contrast, when maximizing structural motif number, we obtained networks with strikingly different structural attributes (Figure 5B) that bore no resemblance to real brain networks. We found no overlap with real networks of significantly enhanced motifs at any of the motif sizes we examined.

To further characterize these networks, we calculated their clustering coefficient and their path length to determine if they exhibited small-world properties (Figure 5). We found that networks that maximized functional motif number also had clustering coefficients that were much higher than those of random networks (γ = 0.5288 ± 0.0201 for optimized networks; γ = 0.4323 ± 0.0073 for random networks), while their path lengths remained relatively short (λ = 1.7891 ± 0.0275 for optimized networks; λ = 1.9300 for a nearest-neighbor lattice network). Both measures closely approximated those of macaque visual cortex (γ = 0.5313, λ = 1.7256). In contrast, networks that maximized structural motif number had clustering coefficients that were indistinguishable from those of random networks (γ = 0.4273 ± 0.0029), and were significantly lower than that of macaque visual cortex.

## Discussion

The importance of a large repertoire of functional circuits for flexible and efficient neural processing has long been recognized (Walter 1953; Ashby 1960) and has recently received renewed theoretical and experimental attention (Tononi et al. 1999; Tononi and Sporns 2003). In this paper we investigate the building blocks of brain networks and how their composition and topological patterning enables flexible neural function. Our hypotheses and analysis rest upon a fundamental distinction between structural and functional motifs. In this work, functional motifs refer to the different patterns or combinations of nodes and connections that could occur within the constraints of a given structural motif. We do not assume anything about their function, or which functional motif is actually selected by physiological mechanisms. We only assume that a particular structural motif is necessary to support a repertoire of functional motifs that may, or may not, be called upon for neuronal computations. Our hypothesis is that the connection patterns of real brain networks maximize functional motif number and diversity, thus ensuring a large repertoire of functional or effective circuits, while they minimize the number and diversity of structural motifs, thus promoting efficient assembly and encoding. We observe that the functional motif number of a variety of real brain networks is very high compared to equivalent random networks, while their structural motif number is comparably low. We then demonstrate that optimization of functional motif number can yield networks that resemble real brain networks in several structural characteristics, including their motif frequency spectra, motifs that occur in significantly increased numbers, and small-world measures.

The functional implications of some network structures—such as reciprocal, convergent, and divergent connections or cycles—have been discussed in the context of network participation indices (Kötter and Stephan 2003) and network complexity (Sporns et al. 2000). Various large-scale cortical connection matrices examined in this study and collected by different authors and from different species, exhibit striking commonalities in their global patterning and motif compositions. Particularly interesting is the increased occurrence of a single motif at *M* = 3 (ID = 9; see Figure 3) and its expanded versions at *M* = 4 (ID = 46, 95, 148, 178). These motifs essentially form of a chain of reciprocally connected units, while pairs of connections linking the ends of the chain are absent. In functional terms, units in these motifs are highly integrated with their neighbors, while some pairs of units remain more segregated from each other and do not communicate directly. Thus, this motif type combines two major principles of cortical functional organization, integration and segregation (Tononi et al. 1998; Friston 2002), and it may be associated with a specific type of neural dynamics (Zhigulin 2003). The occurrence of this motif type is not due to an artifact of recording or collating connection pathways, as it also appears in increased proportion in optimized and rewired networks (see Table 4). In contrast to large-scale cortical networks, the invertebrate network of C. elegans exhibits very different patterns that are less indicative of high integration and segregation. At *M* = 3, motif ID = 9 does not occur in higher-than-expected numbers, while other motifs (ID = 4 and ID = 6) are increased. Our results suggest that large-scale cortical connection matrices form a distinct family (Milo et al. 2004) of networks that can be characterized by their motif frequency spectra, while invertebrate neuronal networks do not appear to belong to this family.

Optimizing functional motif number yields networks that resemble real brain networks across a broad spectrum of structural measures, including several that did not appear to be linked in trivial ways to the optimized measure. Increasing the functional motif number tends to lead to a concomitant decrease in structural motif number, as individual connections become locally dense, thus increasing the abundance of motifs with more local connections and thus greater functional diversity. We note that maximal numbers of functional motifs are not reached in ideal lattices (nearest-neighbor connectivity); rather, optimized networks routinely exhibit functional motif numbers that exceed those of ideal lattices, and they belong to a general class of networks that maintain a mixture of “local” and “long-range” connectivity. Importantly, even though structural and functional motifs are directly related (each structural motif contains a fixed set and spectrum of functional motifs), optimizing structural and functional motif number yielded strikingly different connection topologies.

Optimizing functional (but not structural) motif number produced a tendency toward the emergence of small-world attributes (high clustering coefficient and short path length), a mode of connectivity that promotes functional cooperation, recurrent processing, and efficient information exchange (Sporns et al. 2004). High clustering is due to “locally dense” connectivity promoting fewer, denser, and functionally more potent motifs. An admixture of “long-range” connections, which is compatible with achieving very high functional motif number, serves to maintain short minimal paths throughout the network. Interestingly, networks optimized for complexity (Tononi et al. 1994; Sporns et al. 2000) also exhibit small-world attributes, conserve wiring length, and produce motif frequency spectra similar to those of networks optimized for functional motif number (including a significantly increased abundance of motif ID = 9, *M* = 3; unpublished data). In turn, networks optimized for functional motif number have significantly higher complexity than random networks, while those optimized for structural motif number are much less complex. Thus, it appears that several criteria for optimality (complexity, clustering coefficient, wiring length, functional motif number) favor similar global network architectures that are all characterized by two coexisting organizational principles, functional segregation and functional integration. The functional motif frequency spectrum provides a sophisticated way of characterizing subtypes of such networks geared at more specific functional modes of information processing.

## Materials and Methods

### 

#### Formal definitions

All networks and network motifs in this paper are described as graphs of units (called nodes or vertices) with directed (i.e., nonsymmetrical) connections (called edges).

A “motif” is a connected graph or network consisting of *M* vertices and a set of edges (maximally *M* ^2^ – *M,* for directed graphs, minimally *M* – 1 with connectedness ensured) forming a subgraph of a larger network. For each *M* there is a limited set of distinct motif classes. For *M* = 2, 3, 4, and 5, the corresponding numbers of motif classes are 2, 13, 199, and 9,364 (Harary and Palmer 1973). See Figure 1B for an illustration of the set of 13 motif classes for motifs of size *M* = 3.

A “structural motif” of size *M* is composed of a specific set of *M* vertices that are linked by edges (Figure 1A). The resulting network of size *M* is called a “structural motif” because a larger network could be structurally assembled from a finite set of such motifs. Essentially, structural motifs form the structural building blocks of larger networks. Our definition of structural motifs is identical to the definition of motifs introduced in Milo et al. (2002).

A structural motif provides the complete anatomical substrate for possible functional interactions among its constituent vertices. However, in real neuronal networks, not all structural connections participate in functional interactions at all times. As different edges or connections become functionally engaged, different “functional motifs” emerge within a single structural motif. The former (functional) refers to “processing modes” or “effective circuits,” while the latter (structural) refers to “anatomical elements” or “building blocks.” The existence of different functional motifs greatly enhances the processing power of any neuronal architecture. We then distinguish structural motifs from functional motifs that form a set of subgraphs of the structural motif. All such functional motifs consist of the original *M* vertices of the structural motif, but contain only a subset of its edges (see Figure 1C for examples). Note that a fully connected structural motif such as ID = 13 for *M* = 3 contains the maximal number of functional motifs. For each exemplar of a structural motif of a specific motif class, there is a fixed complement of constituent potential functional motifs (essentially forming a look-up table of potential functional circuits). Thus, the functional motif frequency spectrum is easily obtained from the structural motif frequency spectrum, without the need for additional motif detection.

This definition implies that functional motifs are more naturally applied to networks with vertices that contain multiple neurons or neuronal populations. In the present study, our main focus is on motifs of large-scale connection matrices; data for the single neuron network of C. elegans are provided in Table 1 for statistical comparison only.

A “connected motif” is a structural motif that forms a strongly connected graph. In a connected motif, all constituent vertices can be reached from all other constituent vertices. Such a motif, in principle, allows all vertices to exert causal effects on each other. For *M* = 3, motifs with ID = 7, 9, 10, 12, and 13 are connected motifs.

A “motif frequency spectrum” records the number of occurrences of each motif of a given class for a size *M*. The motif frequency spectrum for structural motifs is obtained by motif detection. The motif frequency spectrum for functional motifs can be obtained from the structural spectrum by simple multiplication with the characteristic number of functional motifs for the respective structural motif.

“Motif number” is the total number of all motifs of all classes (for a given size *M*) encountered in a network. The motif number is obtained as the sum over the motif frequency spectrum, either structural or functional.

“Motif diversity” is the total number of all motif classes (for a given size *M*) encountered in a network. The motif diversity is obtained as the number of all motif classes for which the frequency spectrum is greater than zero.

“Motif participation number” is the number of instances of a given motif class that a particular vertex participates in. For example, if a vertex participates in 12 distinct motifs with *M* = 3, ID = 13, it has a motif participation number of 12 for this particular motif.

The “motif fingerprint” is the spectrum of motif participation numbers for all motifs of a given size *M* that a particular vertex participates in. The motif fingerprint is equivalent to a motif frequency spectrum for a single vertex of the network.

#### Neurobiological data sets.

All datasets used in this study are available in Matlab format at http://www.indiana.edu/~cortex/CCNL.html. Some of the matrices used in this study have been modified to remove areas with few known connections, or areas that are not part of the cerebral cortex. We note, however, that the nature of the data reported in this paper does not critically depend on these small changes, which usually affected only very small subset of the areas and connections. The connection matrix of the macaque visual cortex is based on Felleman et al. (1991), and was modified as follows. The connections of areas {PITd, PIT, PITv}, {CITd, CIT, CITv}, and {STPp, STP, STPa} were consolidated by eliminating PIT, CIT, and STP and assigning their connections to {PITd, PITv}, {CITd, CITv}, and {STPp, STPa}, respectively. Areas MIP and MDP were eliminated due to lack of connectional information. The modified matrix has *N* = 30 and *K* = 311. The connection matrix of the macaque cortex is based on Young (1993). Two areas, HIPP (the hippocampus) and AMYG (the amygdala) were deleted from the matrix, resulting in *N* = 71 and *K* = 746. The connection matrix of cat cortex was transcribed from Scannell et al. (1999). For the large-scale analysis, density information was discarded and all pathways were encoded as either present or absent. For the analysis of intracortical pathways, we discarded the hippocampus and all thalamocortical pathways. The resulting matrix has *N* = 52 and *K* = 820. The connection matrix of C. elegans (White et al. 1986) was retrieved from http://www.wormbase.org and is described at http://elegans.swmed.edu/parts/neurodata_readme.txt. It contains data for the nerve ring and very anterior section of the ventral cord for two individual hermaphrodite worms (JSH, N2U). We used data of all chemical synapses from both individuals, discarding data on gap junctions (electrical synapses), resulting in a matrix of *N* = 197 neurons and *K* = 1,974 directed connections. Other studies used matrices with *N* = 282 (Watts and Strogatz, 1998), *N* = 280 (Milo et al., 2004), or *N* = 252 (Milo et al., 2002). Despite these variations, our results on motifs in C. elegans are consistent with those of these earlier studies.

Currently available datasets are likely to contain errors or missing connections that have not been investigated and do not take into account possible intersubject variability or rank-ordered or graded connection densities or strengths. While these issues have not been addressed systematically, some exploratory analyses suggest that the results reported in this paper are invariant with respect to small variations in connection patterns.

#### Reference cases: random and lattice networks.

A statistical evaluation of motif frequencies depends on a choice of reference cases (“null hypotheses”). Milo et al. (2002) generated random networks with identical structural motif frequencies at level *M* – 1 in order to perform statistical comparisons at level *M*. This corrected for the “carrying over” of significant motif components from lower to higher levels and allowed detection of the level of *M* at which significant structures emerged. The choice of reference cases in this paper reflects the specific question we ask about motifs in brain networks: Independent of the level *M,* how do the motif number, diversity, and composition of real brain networks compare to other network topologies, specifically to both random and lattice networks? We constrain the comparison by fixing the size of the networks *(N* and *K)* and by imposing equal degree distributions on all comparison networks (see also Milo et al. 2004). We note that the additional reference case of the lattice network led to the exclusion of motifs that occur in increased numbers simply because of local clustering of connections (Artzy-Randrup et al. 2004; Milo et al. 2004a).

Random and lattice matrices that preserve the in-degree and out-degree for each vertex are generated from the original anatomical connection matrices by a Markov-chain algorithm (Maslov and Sneppen 2002; Milo et al. 2002). For random matrices, a pair of vertices (*i*_1_,*j*_1_) and (*i*_2_,*j*_2_) is selected for which c*_i_*_1*j*1_ = 1, c*_i_*_2*j*2_ = 1, c*_i_*_1*j*2_ = 0, and c*_i_*_2*j*1_ = 0. Then we set c*_i_*_1*j*1_ = 0, c*_i_*_2*j*2_ = 0, c*_i_*_1*j*2_ = 1, and c*_i_*_2*j*1_ = 1. This is repeated until the connection topology of the original matrix is randomized.

For lattice matrices, the same Markov procedure is employed but swaps are only carried out if the resulting matrix has nonzero entries that are located closer to the main diagonal (thus approximating a lattice or ring topology). This algorithm is implemented as a probabilistic optimization using a weighted cost function.

#### Numerical methods.

All graph theory methods used in this paper—including those for calculating clustering coefficients and path lengths (Sporns 2002)—as well as motif detection algorithms are available in Matlab format at http://www.indiana.edu/~cortex/CCNL.html. In some cases, for large networks or high values of *M,* we employed random sampling to estimate motif frequency spectra and their associated values for motif number and diversity. We selected different sample sizes to ensure convergence of these estimates and performed up to ten separate runs to generate good estimates. The evolutionary algorithms used in this study for optimizing structural and functional motif numbers of networks were similar to the algorithm described in Sporns et al. (2000). Briefly, motif number was calculated for generations of ten individuals. The single individual with the highest motif number was selected and copied; all other individuals were deleted. The next generation was composed of the single survivor and nine rewired copies (using a rewiring rate of one connection). The first generation was composed of ten random networks. The rewiring procedure typically proceeded for 2,000 generations, changing only the connection pattern or topology. *N, K,* and the original degree distribution were conserved.

## Abbreviations

ID: motif identity number
*K*: number of edges
*M*: motif size
*N*: number of vertices
